# Neu5Gc and beyond: sialylation at the innate immune and thromboinflammatory interface of xenotransplantation

**DOI:** 10.3389/frtra.2026.1968710

**Published:** 2026-09-28

**Authors:** Jae Young Kim

**Affiliations:** Department of Life Science, Gachon University, Seongnam, Kyeonggi-Do, Republic of Korea

**Keywords:** endothelial glycocalyx, glycoengineering, innate immunity, Neu5Gc, sialic acid, Siglec, thromboinflammation, xenotransplantation

## Abstract

Recent advances in genetic engineering have substantially reduced the major carbohydrate barriers to pig-to-human xenotransplantation. Deletion of GGTA1, CMAH, and B4GALNT2 decreases *α*Gal, Neu5Gc, and B4GALNT2-dependent Sda-like xenoantigens and thereby attenuates preformed antibody binding and complement-mediated injury. However, removal of these major xenoantigens does not necessarily generate a human-compatible cell-surface glycome. In particular, CMAH knockout eliminates Neu5Gc but leaves a Neu5Ac-dominant sialome whose linkage, chemical modification, glycoconjugate carrier, density, and spatial presentation are not necessarily human-like. This review examines sialylation and glycan-dependent recognition as an underappreciated regulatory layer linking innate immunity, blood-cell homeostasis, complement regulation, and thromboinflammation in xenotransplantation. Two conceptual distinctions are emphasized. First, the direction and evidence depth of glycan–receptor recognition must be explicitly considered. The best-established Siglec-1-dependent xenogeneic interaction is a graft-versus-host pathway, in which donor porcine Siglec-1-positive macrophages capture recipient human erythrocytes. Conversely, direct receptor-level binding of human Siglec-1 to CMAH-deficient gene-edited pig erythrocytes has recently been demonstrated, providing evidence for the reciprocal host-versus-graft direction; however, whether this interaction drives recipient macrophage phagocytosis or *in vivo* clearance remains unresolved. Donor ASGR1-dependent uptake of human platelets provides another direct example of glycan-dependent recipient blood-cell clearance. Second, *α*2,3-linked Neu5Ac illustrates a fundamental functional trade-off in sialome engineering. Neu5Ac*α*2,3Gal-containing glycans can serve as preferred ligands for Siglec-1-mediated capture, whereas *α*2,3-linked sialoglycans also contribute to Factor H-dependent recognition of self-associated surfaces and alternative complement regulation. Conversely, increasing *α*2,6-linked sialylation may reduce macrophage xenoreactivity and enhance inhibitory Siglec engagement but decrease *α*2,3-dependent Factor H recognition; thus, ST6GAL1 engineering should not be considered uniformly protective or equivalent to glycan “humanization”. Other pathways, including CD24–Siglec-10 signaling, Siglec-9-mediated regulation of neutrophil activation and NETosis, and platelet Siglec signaling, provide additional mechanistic frameworks but remain incompletely validated in solid-organ xenotransplantation. We therefore propose that post-editing glycan compatibility should be evaluated according to receptor directionality, evidence depth, and functional trade-offs, rather than glycan abundance or donor genotype alone. Organ-, cell-, carrier-, and receptor-specific analyses integrating quantitative glycomics/glycoproteomics, human receptor and immune-cell assays, functional crossmatching, and *ex vivo* perfusion are needed to link donor genotype to post-editing glycan phenotype and guide precision optimization of xenograft compatibility.

## 1 Introduction

Organ transplantation is the only definitive treatment for end-stage organ failure, yet the shortage of transplantable human organs remains a major clinical challenge. Pigs are currently considered the most practical donor species for xenotransplantation because of their organ size and physiological characteristics similar to those of humans, rapid breeding, and amenability to genetic modification (1, 2).

Early pig-to-primate organ transplantation was limited by hyperacute rejection, which occurs when naturally occurring recipient antibodies recognize major carbohydrate xenoantigens on porcine vascular endothelium and activate complement. The development of triple-knockout (TKO) pigs lacking the major xenoantigens *α*Gal, Neu5Gc, and the B4GALNT2-dependent Sda-like glycan substantially reduced this antibody barrier (3–5). In parallel, the introduction of human genes regulating complement, coagulation, innate immunity, and cytoprotection has markedly prolonged xenograft survival in non-human primates (6).

These advances led to important proof-of-concept studies in humans beginning in 2022. Porrett et al. transplanted a gene-edited pig kidney into a human decedent model and demonstrated short-term graft function without hyperacute rejection (7). In the same year, Griffith et al. orthotopically transplanted a multi-gene-edited pig heart into a patient with end-stage heart failure, where the graft provided life-sustaining function for approximately two months (8). Subsequent analyses of graft dysfunction suggested a multifactorial process involving endothelial injury, antibody-mediated immune activation, and infection-related factors, indicating that vascular, complement, and thromboinflammatory barriers may persist even after removal of major xenoantigens (9). In 2024, a gene-edited pig kidney was transplanted into a living recipient, with the clinical course subsequently reported (10), and in 2025, a gene-edited pig liver maintained short-term metabolic and bile-secretory functions in a brain-dead human recipient (11). These cases demonstrate the clinical feasibility of xenotransplantation while also underscoring the need for a more precise understanding of the residual immunological and hemocompatibility barriers that remain after genetic engineering.

Indeed, antibody-mediated vascular injury, complement activation, thrombotic microangiopathy, platelet consumption, and innate immune-cell activation are not completely eliminated even in donor organs incorporating TKO and multiple human transgenes. This suggests that xenograft injury is not determined by a single antigen or immune pathway, but rather by a complex process in which antibody–complement responses, coagulation incompatibility, endothelial activation, and innate cellular recognition mutually amplify one another. Therefore, removal of major xenoantigens should not be equated with complete immunological humanization of the donor organ.

In this context, one interspecies difference that remains insufficiently evaluated is the sialome. In this review, the sialome is defined as the entire repertoire of sialic acid-containing glycoconjugates present in a given cell or tissue, encompassing not only the type of sialic acid but also *α*2,3-, *α*2,6-, and *α*2,8-glycosidic linkages, chemical modifications, underlying glycan structures, glycoprotein or glycolipid carriers, surface density, and spatial presentation (12, 13). Although CMAH knockout reduces the important anti-Neu5Gc antibody barrier, removal of Neu5Gc does not necessarily generate a human-like functional sialome (14).

Previous major reviews have focused primarily on Neu5Gc and the anti-Neu5Gc barrier (14), non-Gal xenoantigens including B4GALNT2-dependent glycans (5), the structural and functional diversity of sialic acids (13), or the broader immunological barriers to xenotransplantation (15). Building on these studies, the present review highlights two interrelated conceptual issues. First, glycan–receptor interactions should be interpreted not only in terms of the presence of receptors and ligands but also with explicit consideration of the directionality of recognition. Second, because the same sialoglycan can exert both detrimental and protective effects through different receptor systems, glycoengineering is inherently subject to structural and functional trade-offs.

Accordingly, the central question addressed in this review is not simply which glycans differ between pigs and humans, but rather which receptors recognize specific structures within the post-editing sialome, in which direction these interactions occur, and how their beneficial and detrimental consequences are balanced in actual xenogeneic outcomes. To address this question, we first review the major glycan and sialome differences between pigs and humans and the residual structural gaps that remain after TKO. We then evaluate glycan-dependent macrophage, neutrophil, and platelet responses, together with Factor H-mediated complement regulation, while distinguishing direct xenotransplantation evidence from intermediate mechanistic evidence and as-yet-unvalidated hypotheses. Finally, we discuss future donor-engineering and functional-validation strategies from the perspective of glyco-immune compatibility, integrating organ- and cell-restricted glycoengineering, carrier-level modification, sialidase inhibition and glycocalyx preservation, and complement/coagulation control, with explicit consideration of receptor directionality and functional trade-offs.

## 2 Glycan differences between pigs and primates, with emphasis on the sialic acid profile

### 2.1 Major glycan xenoantigens relevant to xenotransplantation

The best-established carbohydrate xenoantigens in pig-to-human xenotransplantation are galactose-α1,3-galactose (*α*Gal), N-glycolylneuraminic acid (Neu5Gc), and the porcine B4GALNT2-dependent Sda-like glycan, which are associated with GGTA1, CMAH, and B4GALNT2, respectively (4, 5, 14). Naturally occurring or induced human antibodies against these glycans can promote antibody binding, complement activation, and vascular endothelial injury, making them major targets of donor genetic engineering.

*α*Gal, with the structure Gal*α*1-3Gal*β*1-4GlcNAc-R, is widely expressed on glycoproteins and glycolipids of porcine cells. Humans and Old World monkeys do not express functional GGTA1 and therefore cannot synthesize *α*Gal, but they possess naturally occurring anti-αGal antibodies (5, 16). Although GGTA1 knockout (GTKO) markedly reduced this antibody barrier and the risk of hyperacute rejection, antibody responses to non-Gal antigens, including Neu5Gc and B4GALNT2-dependent glycans, remain after GTKO. This led to the development of GGTA1/CMAH/B4GALNT2 triple-knockout (TKO) pigs, in which CMAH and B4GALNT2 are additionally deleted. TKO cells show lower IgM and IgG binding than WT or single- and double-knockout cells in most human sera (4, 17). However, elimination of these three major xenoantigens does not convert the entire porcine glycome into a human-like state, and residual glycans as well as post-editing glycome remodeling must still be considered.

### 2.2 Human–pig sialome mismatch: structural differences beyond Neu5Gc

Humans lost functional CMAH during evolution and therefore cannot endogenously synthesize Neu5Gc; consequently, Neu5Ac is the predominant sialic acid on cell-surface glycoconjugates (18–20). In contrast, wild-type pigs broadly express Neu5Gc-containing glycoproteins and glycolipids (21, 22). Accordingly, although substantial interindividual variation exists, human sera contain appreciable levels of anti-Neu5Gc antibodies (23, 24).

The consequences of CMAH deletion are particularly complex in non-human primate models. Unlike humans, Old World monkeys, including baboons and macaques, retain functional CMAH, and some Old World monkeys exhibit naturally occurring antibodies against TKO pig cells that are rarely observed in humans (25). These differences raise the possibility that other carbohydrate structures exposed or relatively increased on Neu5Gc-deficient pig cells may be recognized as non-self in NHP recipients. Indeed, in a baboon kidney xenotransplantation study comparing Neu5Gc-expressing GTKO kidneys with Neu5Gc-negative DKO/TKO kidneys, the latter showed shorter graft survival and more frequent histological features of antibody-mediated rejection (26). Although these findings do not directly demonstrate the generation of a specific neo-glycan or establish an isolated effect of CMAH knockout, they provide direct experimental evidence that the immunological consequences of xenoantigen deletion can vary according to the recipient species and that CMAH knockout should not be interpreted simply as sialome humanization.

The function of sialic acids is determined not only by whether the terminal residue is Neu5Ac or Neu5Gc, but also by *α*2,3-, *α*2,6-, and *α*2,8-linkages, the underlying glycan structure, chemical modifications, and the glycoprotein or glycolipid carrier (13). Because CMAH determines the type of sialic acid rather than catalyzing the formation of glycosidic linkages, the linkage patterns and cell-surface presentation of Neu5Ac that increases after CMAH knockout are determined by the intrinsic glycosylation machinery of porcine tissues. Thus, specific Neu5Ac-containing glycans may become relatively enriched or more exposed after CMAH knockout, and such changes may alter recognition by glycan-binding receptors such as Siglecs independently of the reduction in antibody reactivity. Therefore, the consequences of CMAH knockout should be evaluated not only in terms of Neu5Gc elimination but also from the perspective of the post-editing functional sialome, which may vary according to recipient species and donor organ.

### 2.3 B4GALNT2-dependent Sda-like glycan: a major non-Gal antigen and post-editing precursor changes

The Sda-related glycan generated by B4GALNT2 is one of the major non-Gal xenoantigens that remains relevant after the removal of *α*Gal and Neu5Gc (5, 27). A representative terminal structure is GalNAc*β*1-4(Neu5Ac*α*2-3)Gal*β*-R, in which B4GALNT2 adds GalNAc in a *β*1,4 linkage to an *α*2,3-sialylated galactose-containing precursor. Thus, this antigen is not a structure independent of sialic acid, but rather a branched glycan formed on an *α*2,3-sialylated precursor (5). Importantly, humans also express functional B4GALNT2, and its enzymatic product, Sda [GalNAc*β*1-4(Neu5Ac*α*2-3)Gal*β*-R], constitutes part of the human SID histo-blood group system. However, Sda expression in humans is highly quantitative and tissue dependent. Indeed, B4GALNT2 is polymorphic in humans, and missense variants in the C-terminal portion of the encoded glycosyltransferase underlie the Sd(a−) phenotype (28). Conventional serologic studies detect anti-Sda IgM in only a small fraction of individuals, whereas erythrocytes with unusually high Sda expression, such as the CAD/Sd(a++) phenotype, reveal low-level anti-Sda IgM reactivity in a much larger proportion of human sera (5). Thus, endogenous Sda expression does not imply complete immunological tolerance to all quantitative or contextual presentations of B4GALNT2-dependent glycans. In pigs, broad vascular endothelial expression of B4GALNT2, together with species-dependent differences in acceptor glycans, glycoconjugate carriers, and epitope density, may generate an antigenic surface distinct from the generally lower and tissue-restricted human Sda presentation. Accordingly, porcine B4GALNT2-dependent xenoantigenicity should not be equated simply with the presence or absence of the canonical human Sda trisaccharide (5). Indeed, B4GALNT2-dependent glycans can become targets of non-Gal antibodies generated after pig-to-NHP transplantation, and B4GALNT2 knockout further reduces human IgM and IgG binding to GGTA1/CMAH double-knockout cells (4, 5).

The significance of B4GALNT2 knockout may extend beyond removal of the Sda-like antigen itself. Because B4GALNT2 uses an *α*2,3-sialylated precursor as its substrate, deletion of the enzyme may result in relative retention or increased exposure of this precursor. Whether such changes increase binding by Siglec-1 or other *α*2,3-sialoglycan-binding receptors has not yet been directly demonstrated. Nevertheless, this possibility highlights an important principle: glycan editing may simultaneously remove a target antigen while altering the availability of ligands for other glycan-binding receptors. Accordingly, B4GALNT2 knockout should be interpreted not only in terms of its established benefit in reducing antibody binding, but also in the context of residual *α*2,3-sialylated precursors and broader post-editing glycome remodeling.

### 2.4 Organ- and cell type-specific glycome heterogeneity and residual antigenicity

Even among pigs with the same TKO genotype, residual antigenicity may vary across organs and cell types. Indeed, in TKO pigs lacking *α*Gal, Neu5Gc, and B4GALNT2-dependent glycans, the extent of reduction in human IgM and IgG binding was not uniform among the heart, kidney, lung, liver, spleen, and pancreas (29). This indicates that elimination of the major xenoantigens alone is insufficient to predict the overall immunogenicity of individual organs and that the distribution of other residual glycan or protein antigens may differ among tissues.

Such differences are strongly influenced by organ-specific cellular composition and glycosylation machinery. Because different cell types express distinct repertoires of glycosyltransferases and glycosidases, the same genetic modification may generate different glycomes depending on the cellular context (29, 30). Particularly during the early phase after transplantation, the glycome of organ-specific microvascular endothelium, which is directly exposed to recipient blood, may be an important determinant of antibody binding, complement activation, and interactions with innate immune cells. Therefore, findings obtained using surrogate cells, such as fibroblasts or aortic endothelial cells, cannot be assumed to fully represent the microvascular glycome of the actual transplanted organ.

Differences in glycoconjugate carriers may also determine functional antigenicity. Even when the same terminal glycan structure is present, its accessibility to antibodies and lectin receptors can vary according to the type of glycoprotein or glycolipid carrier, surface density, and multivalent presentation (31). Moreover, glycosyltransferase knockout does not simply remove the targeted antigen; it may redirect unused precursors into alternative biosynthetic pathways, resulting in relative enrichment of pre-existing glycans or exposure of new terminal structures (31, 32). Thus, the effects of gene editing should not be assumed to increase simply in proportion to the number of knockout genes, but should instead be evaluated on the basis of the actual glycome and residual antigenicity of each target organ.

### 2.5 Limitations of TKO and assessment of functional compatibility

Triple-knockout (TKO) pigs lacking GGTA1, CMAH, and B4GALNT2 show substantial reductions in the three best-established major carbohydrate xenoantigens and markedly decreased IgM/IgG binding to porcine cells in sera from many human individuals (4, 29, 33). However, TKO cells may still retain protein antigens, including SLA class I, as well as incompletely characterized residual glycan antigens, and responses to these remaining antigens may persist in some sensitized recipients (17, 34).

As discussed above, glycan editing can remodel the residual glycome and sialome while eliminating the targeted antigens, and the resulting changes may vary among organs and cell types. Therefore, the compatibility of TKO donors should not be assessed solely on the basis of the absence of *α*Gal, Neu5Gc, and B4GALNT2-dependent glycans. Rather, glycans that remain or become relatively enriched after editing, together with their functional interactions with the human immune system, should also be evaluated.

Several complementary approaches will be required. First, human IgM/IgG binding and donor-specific crossmatching should be assessed using microvascular endothelial cells and major parenchymal cells derived from the actual target organ. Second, the post-editing glycome should be characterized by quantifying Neu5Ac and Neu5Gc and by profiling *α*2,3-, *α*2,6-, and *α*2,8-linked sialylation, major chemical modifications, and glycoprotein- and glycolipid-associated glycans (31, 35). Third, binding of major glycan-recognition receptors, including Siglecs, should be examined together with macrophage, neutrophil, and NK-cell responses before and after receptor blockade to determine whether structural glycan changes translate into functional alterations in innate immunity. Finally, complement-dependent cytotoxicity, phagocytosis, and other functional assays using recipient serum and blood cells may help define donor–recipient-specific residual risks.

In conclusion, TKO remains a key donor platform for clinical xenotransplantation, but future evaluation of donor pigs should move beyond genotype or major xenoantigen negativity to include organ-specific glycome/sialome profiling and functional compatibility testing using human antibodies, complement, and innate immune cells. Figure 1 conceptually summarizes the transition from major xenoantigen removal to the post-editing sialome and emphasizes that, although TKO is effective in eliminating major carbohydrate antigens, it does not necessarily generate a human-compatible functional sialome.

**Figure 1:**
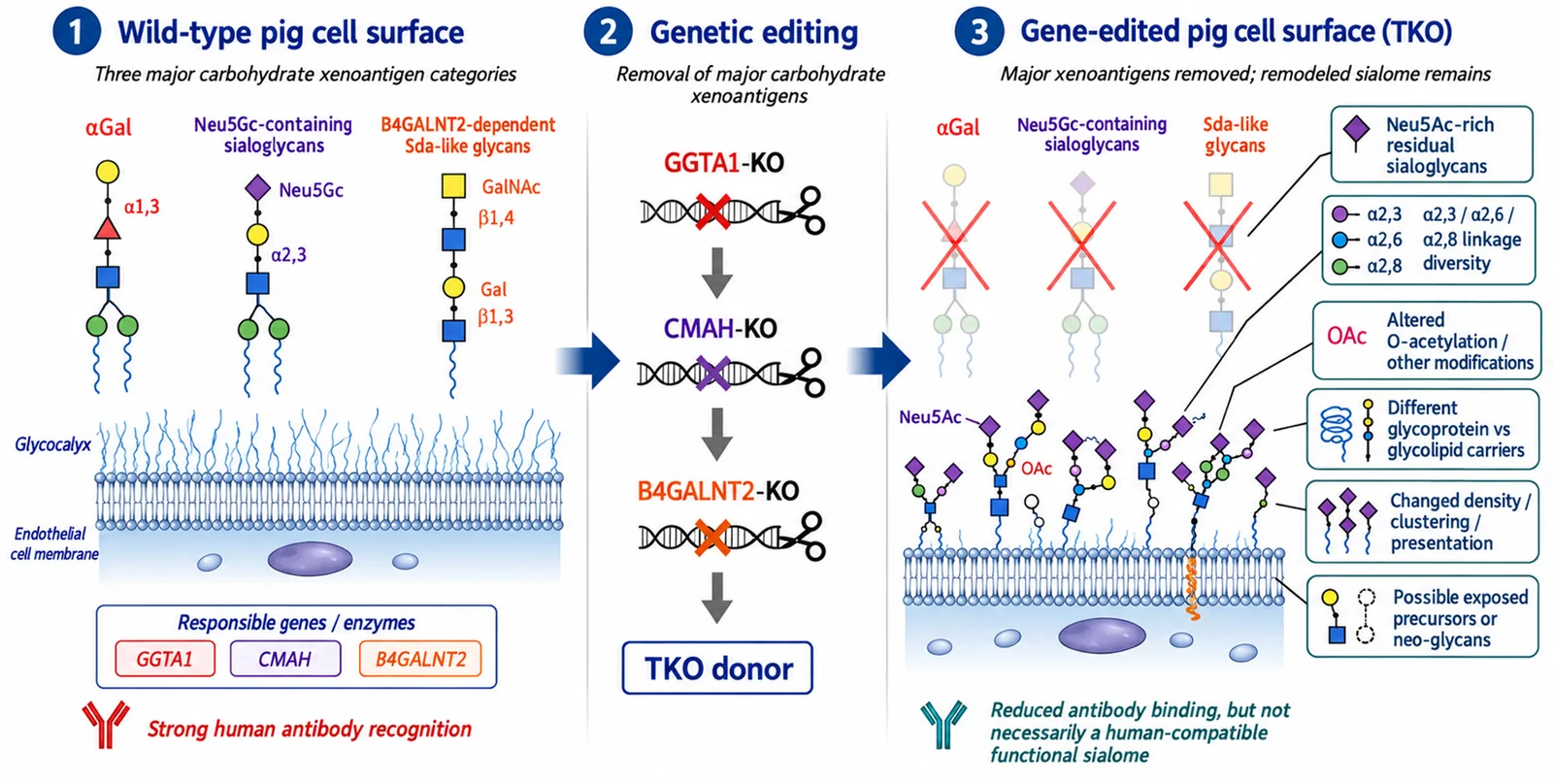
From xenoantigen removal to a remodeled sialome. Wild-type pig cells express three major carbohydrate xenoantigen classes, including *α*Gal, Neu5Gc-containing sialoglycans, and porcine B4GALNT2-dependent Sda-like glycans, which are primarily associated with GGTA1, CMAH, and B4GALNT2, respectively. Genetic disruption of these three genes generates triple-knockout (TKO) pigs and markedly reduces recognition by preformed human antibodies. However, removal of the major xenoantigens does not necessarily generate a human-compatible functional sialome. Following gene editing, residual cell-surface glycans may differ in Neu5Ac abundance, *α*2,3/*α*2,6/*α*2,8 linkage distribution, chemical modifications such as O-acetylation, glycoprotein versus glycolipid carriers, surface density and clustering, and exposure of precursor or neo-glycan structures. These post-editing changes may alter recognition by glycan-binding receptors, including Siglecs, and potentially affect Factor H-dependent complement regulation. Importantly, the remodeled glycan features illustrated in the TKO panel represent potential consequences of glycosyltransferase editing and should not be interpreted as uniformly established changes across all TKO pig tissues. Thus, assessment of genetically engineered donor pigs should extend beyond loss of *α*Gal, Neu5Gc, and Sda-like antigens to include organ- and cell-specific characterization of the residual functional glycome and sialome.

## 3 Potential inflammatory and coagulation responses mediated by the sialic acid–Siglec axis

### 3.1 Overview: inhibitory and activating Siglec signaling and sialic acid recognition

Siglecs (sialic acid-binding immunoglobulin-like lectins) are a family of receptors that recognize sialylated glycans and regulate activation thresholds in immune cells (36, 37). Most CD33-related Siglecs recruit SHP-1/2 through ITIM or ITIM-like motifs and thereby suppress immune-cell activation, whereas activating CD33-related Siglecs such as Siglec-14 and Siglec-16 associate with DAP12 and signal through ITAM-dependent pathways. Siglec-15 is not a CD33-related Siglec but an evolutionarily conserved member of the Siglec family; nevertheless, it can associate with activating adaptor proteins, including DAP12, through a charged residue in its transmembrane domain (38). Paired receptors, such as Siglec-5 and Siglec-14, can exhibit similar ligand-binding specificities while delivering opposing signals (37, 39). In contrast, Siglec-1 (CD169/sialoadhesin) lacks a canonical ITIM or DAP12-coupling motif and primarily mediates the adhesion and capture of sialylated targets (36).

Siglec ligand recognition is determined not only by the type and linkage of sialic acid but also by the underlying glycan structure, chemical modifications, glycoconjugate carrier, surface density, and multivalent presentation (40). The crystal structure of the N-terminal V-set domain of Siglec-1 in complex with 3′-sialyllactose directly demonstrated the structural basis for recognition of Neu5Ac*α*2,3Gal-containing ligands (41), whereas actual binding at the cell surface can vary substantially depending on the carrier and multivalent presentation of these glycans (42).

Because of these features, replacement of Neu5Gc with Neu5Ac after CMAH knockout does not automatically generate a human-like Siglec ligand landscape. Post-editing porcine sialoglycans may fail to provide sufficient engagement of inhibitory Siglecs in the recipient or, conversely, may enhance interactions with particular activating Siglecs or capture receptors. However, direct evidence that such Siglec under-engagement or mis-engagement drives rejection in solid-organ xenotransplantation remains limited. Accordingly, the following sections distinguish direct xenotransplantation evidence from mechanistic evidence derived from other experimental systems when evaluating each pathway. The major glycan-dependent recognition pathways discussed in this section, together with their respective levels of evidence, are conceptually summarized in Figure 2. Importantly, the figure distinguishes donor glycan–recipient receptor/regulator pathways from donor receptor–recipient glycan pathways, because only the former are direct consequences or potential targets of donor sialome remodeling.

**Figure 2:**
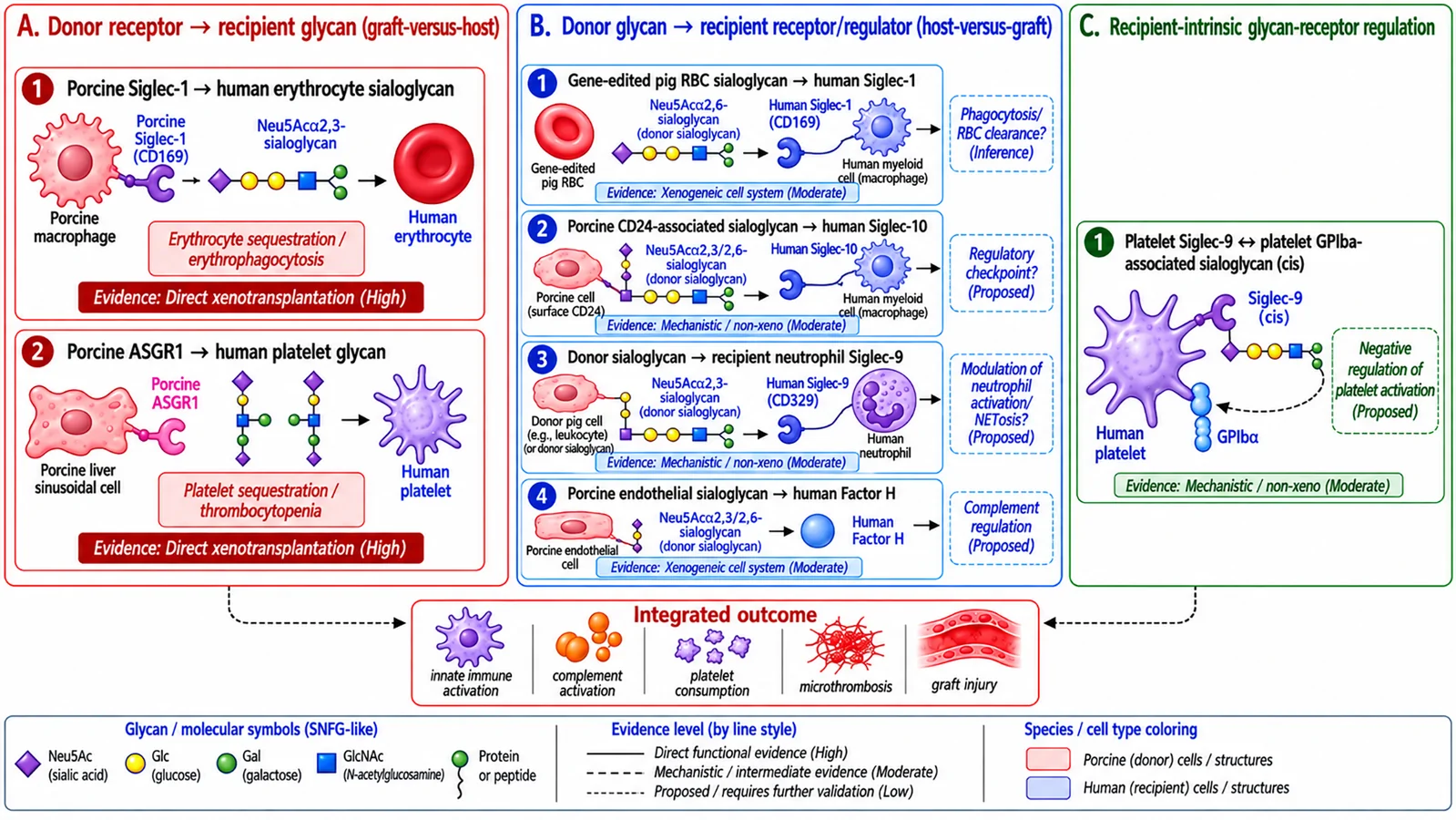
Directionality and evidence levels of glycan-dependent innate immune and thromboinflammatory pathways relevant to xenotransplantation. The schematic distinguishes three mechanistic contexts. Donor receptor–recipient glycan (graft-versus-host) pathways include porcine Siglec-1-mediated capture of human erythrocytes and donor ASGR1-dependent human platelet clearance, both of which are supported by direct xenotransplantation evidence. Donor glycan–recipient receptor/regulator (host-versus-graft) pathways include direct receptor-level binding of human Siglec-1 to gene-edited pig RBCs and candidate interactions of donor sialoglycans with recipient Siglecs or Factor H. Recipient-intrinsic glycan–receptor pathways, including platelet Siglec signaling, provide additional mechanistic context but are not themselves direct consequences of donor sialome remodeling. Thus, the figure represents an evidence-weighted framework rather than a single causal cascade or a set of pathways uniformly attributable to donor sialome remodeling. Solid lines indicate direct functional xenotransplantation evidence, dashed lines indicate mechanistic or intermediate evidence, and dotted lines indicate proposed relationships requiring direct validation.

### 3.2 Suppression of DAMP-driven inflammation through the CD24–Siglec-10 innate immune checkpoint

The CD24–Siglec-G/10 axis functions as an innate immune checkpoint that restrains excessive inflammatory responses to damage-associated molecular patterns (DAMPs), including HMGB1 and HSP70/90, released during tissue injury (43, 44). Siglec-G mediates this function in mice, whereas Siglec-10 serves as its functional counterpart in humans. ITIM-dependent signaling through Siglec-10 recruits SHP-1/2 and limits DAMP-associated inflammatory signaling. CD24 is a highly glycosylated GPI-anchored protein, and its sialylation plays an important role in its interaction with Siglec-10 (45).

In xenotransplantation, DAMPs are released during organ procurement, ischemia, and reperfusion, raising the possibility that insufficient inhibitory Siglec signaling could amplify early sterile inflammation. In particular, if the structure or surface presentation of porcine CD24-associated sialoglycans is suboptimal for recognition by human Siglec-10, inhibitory signaling in recipient macrophages and dendritic cells may be attenuated, thereby enhancing DAMP-induced inflammatory responses. Findings from other disease models showing that the CD24–Siglec-10 axis can function as a macrophage inhibitory checkpoint independently of CD47–SIRP*α* support the biological plausibility of this mechanism (46).

### 3.3 Inhibitory Siglecs on macrophages and neutrophils and their link to NETosis–thromboinflammation

Inhibitory CD33-related Siglecs, including Siglec-3, -5, -9, and -10, are expressed on macrophages and neutrophils and limit signaling through TLRs, Fc receptors, and other activating receptors via ITIM-dependent pathways (37). Therefore, insufficient engagement of these inhibitory Siglecs by graft sialoglycans may lower the activation threshold for cytokine production, oxidative burst, degranulation, and phagocytosis. However, because individual Siglecs differ in their cellular distribution and ligand specificity, and because paired receptors such as Siglec-5/14 can transmit opposing signals, it is not appropriate to assume that increased sialylation necessarily results in stronger suppression of innate immune responses.

Siglec-9 is an inhibitory receptor that is particularly important in limiting excessive neutrophil activation. Sialoglycoproteins on normal human erythrocytes suppress the neutrophil oxidative burst through Siglec-9 (47), and synthetic multivalent Siglec-9 agonists can reduce inflammatory stimulus-induced neutrophil activation and NETosis (48). These findings indicate that appropriate Siglec-9 signaling may serve as an important checkpoint restraining excessive neutrophil activation.

Neutrophil extracellular traps (NETs) are composed of extracellular DNA, histones, and neutrophil granule proteins and can amplify thromboinflammation by promoting endothelial injury and the accumulation of platelets and coagulation factors (49). Accordingly, in xenotransplantation, NETosis triggered by antibody/complement activation and ischemia–reperfusion injury may exacerbate platelet deposition and microvascular thrombosis. Indeed, studies showing that porcine cells expressing human CD31 or CD177 reduce human neutrophil NETosis and xenogeneic cytotoxicity support the concept that restoration of appropriate inhibitory signaling can mitigate xenogeneic neutrophil-mediated injury (50, 51).

### 3.4 Platelet perspective: sialylation-dependent homeostasis and Siglec-7/9

Cell-surface sialic acids are important components of the platelet glycocalyx that regulate platelet lifespan and activation state (52). During aging or inflammation, platelet desialylation exposes underlying galactose/GalNAc residues, leading to hepatic clearance through the Ashwell–Morell receptor (ASGR1/ASGR2) (53). Excessive desialylation can therefore contribute to thrombocytopenia and, under certain conditions, may be accompanied by increased activation of the remaining platelets (54, 55).

Siglec-7 can translocate to the platelet surface following activation, and ligand-mediated crosslinking has been reported to induce phosphatidylserine exposure and microparticle formation, indicating that Siglec-7 cannot be viewed simply as an inhibitory checkpoint in platelets (56). In contrast, recent evidence suggests that a cis interaction between *α*2,3-linked sialoglycans on GPIb*α* and platelet Siglec-9 may function as an inhibitory checkpoint that restrains platelet activation (57). These findings raise the possibility that desialylation not only promotes platelet clearance but may also weaken certain inhibitory glycan–receptor interactions.

The most direct evidence for glycan-dependent platelet recognition in xenotransplantation has been obtained in the context of the porcine liver. During *ex vivo* pig liver perfusion, human platelets are rapidly captured and cleared, with lectin-dependent recognition by liver sinusoidal endothelial cells and Kupffer cells contributing to this process (58, 59). In particular, deletion of donor ASGR1 reduces human platelet consumption, demonstrating that glycan-dependent platelet clearance can directly contribute to thrombocytopenia in liver xenotransplantation (60). Therefore, the potential roles of platelet sialylation and the Siglec axis should be distinguished from the established lectin-mediated platelet-clearance mechanisms in the liver, and their functional significance in xenotransplantation requires further validation. Moreover, these mechanisms are organ-specific, and the ASGR1-dependent pathway demonstrated in the liver should not be directly generalized to heart or kidney xenotransplantation.

### 3.5 Siglec-1–sialic acid-dependent recognition and erythrocyte–macrophage interactions

The interaction between porcine Siglec-1 (CD169/sialoadhesin) and human erythrocytes represents one of the clearest examples of directly demonstrated sialic acid-dependent recognition in xenotransplantation. Siglec-1 is expressed on porcine Kupffer cells and alveolar macrophages and recognizes Neu5Ac-rich sialoglycans on human erythrocytes, particularly the *α*2,3-linked structures preferred by Siglec-1, thereby promoting erythrocyte adhesion and phagocytosis (61). This represents a graft-versus-host interaction in which donor porcine macrophages remove recipient human erythrocytes and should be distinguished from host-versus-graft recognition, in which recipient macrophages recognize and eliminate porcine cells.

Brock et al. demonstrated that binding between porcine macrophages and human erythrocytes was markedly reduced by either an anti-porcine Siglec-1 antibody or sialidase treatment of human erythrocytes (61). Moreover, human erythrocyte sequestration and the associated decrease in hematocrit during extracorporeal porcine liver perfusion were partially inhibited by an anti-Siglec-1 antibody, suggesting that this interaction contributes not merely to *in vitro* glycan binding but also to blood-cell consumption at the organ level (62).

An important species difference exists in this pathway. Whereas human erythrocytes are Neu5Ac-dominant because of the absence of functional CMAH, erythrocytes from Old World monkeys, including baboons and macaques, express Neu5Gc and exhibit relatively weaker binding to porcine Siglec-1 (63). Thus, pig-to-NHP models may underestimate Siglec-1-dependent erythrocyte sequestration that could occur in pig-to-human transplantation. Residual binding of human erythrocytes has also been observed in macrophages derived from pigs carrying TKO and multiple human transgenes, and this binding was substantially reduced by either anti-Siglec-1 antibody treatment or sialidase treatment of the erythrocytes (64). However, because these studies used pigs with multiple genetic modifications, the observed effects cannot be attributed to CMAH knockout alone.

Collectively, these findings indicate that Siglec-1-mediated xenogeneic recognition may occur in both directions and that the depth of evidence for each direction should be distinguished. The graft-versus-host pathway, in which porcine Siglec-1 recognizes and sequesters recipient human erythrocytes, has been directly demonstrated in macrophage-based and *ex vivo* xenogeneic systems (61, 62, 64). Conversely, recent receptor–cell-level evidence has shown that recombinant human Siglec-1 directly binds CMAH-deficient gene-edited pig RBCs, including DKO, TKO, and QKO RBCs, and that these cells exhibit greater Siglec-1 binding than WT pig RBCs or human RBCs (65). The marked reduction in binding after sialidase treatment and its competitive inhibition by free Neu5Ac further demonstrate that the interaction between gene-edited pig RBCs and human Siglec-1 is sialic acid-dependent. Notably, although human RBCs contained the highest total Neu5Ac levels, they exhibited lower human Siglec-1 binding than gene-edited pig RBCs (65). This apparent discordance is mechanistically plausible because Siglec-1 does not recognize Neu5Ac indiscriminately: it preferentially binds *α*2,3-linked Neu5Ac-containing motifs, while cell-surface avidity is further influenced by the underlying glycan structure, glycoconjugate carrier, ligand accessibility, surface density, and multivalent clustering. Thus, a cell with less total Neu5Ac may bind Siglec-1 more strongly if a greater fraction of its sialic acids is presented as accessible, multivalent Siglec-1 ligands. However, Pham et al. did not directly resolve the linkage- and carrier-specific glycome of these RBCs; therefore, the molecular basis of the enhanced binding remains to be established by linkage- and carrier-resolved glycomic and glycoproteomic analyses. These findings establish receptor-level host-versus-graft recognition but do not yet demonstrate recipient macrophage effector function or *in vivo* clearance.

### 3.6 Intersection of complement, coagulation, and sialic acid: Factor H and the endothelial glycocalyx

Factor H is a key plasma regulator of the alternative complement pathway. It promotes decay of the C3 convertase and factor I-dependent inactivation of C3b while protecting host cell surfaces through simultaneous recognition of C3b and polyanionic self-associated structures (66, 67). The C-terminal CCP19–20 region is critical for this self-surface recognition, and the sialic acid-binding site located particularly within CCP20 preferentially recognizes *α*2,3-linked Neu5Ac-containing glycans. Structural and biophysical analyses have shown that Factor H binds *α*2,3-linked Neu5Ac but exhibits little or no significant binding to *α*2,6- or *α*2,8-linked Neu5Ac (66, 67). Thus, Factor H-mediated self-recognition depends on specific glycosidic linkages and modes of surface presentation rather than simply on total sialic acid abundance.

This linkage selectivity introduces an important trade-off when interpreting the post-editing sialome. *α*2,3-linked sialoglycans can serve as preferred ligands for Siglec-1 while also functioning as self-associated glycans that support Factor H recruitment. Therefore, uniformly increasing or decreasing a particular sialic acid linkage cannot be assumed to be universally beneficial. Moreover, because Factor H can recognize both Neu5Ac- and Neu5Gc-containing glycans, the major complement-related benefit of CMAH knockout is more appropriately attributed to elimination of the anti-Neu5Gc antibody target rather than to enhanced Factor H binding (68).

Factor H-dependent protection is not determined by sialic acid alone. Glycosaminoglycans, including heparan sulfate, and other polyanionic components of the endothelial glycocalyx also contribute to Factor H recruitment to cell surfaces and regulation of the alternative pathway. In a study using human endothelial cells, reduced glycocalyx density impaired surface Factor H binding and cofactor activity, resulting in dysregulation of the alternative complement pathway and demonstrating a direct functional link between glycocalyx damage and complement-mediated endothelial injury (69). It is therefore plausible that glycocalyx injury occurring during xenograft reperfusion may similarly compromise Factor H-dependent complement control. However, because the study by Teoh et al. was performed in a model of cyclosporine-associated endothelial injury, the extent to which this mechanism operates in xenotransplantation requires direct validation.

Indeed, a comparative study of GTKO and TKO porcine cells found no major difference in C3 deposition or Factor H-mediated regulation under alternative pathway conditions, suggesting that Neu5Gc removal itself does not substantially alter human Factor H function (68). By contrast, the reduction in overall C3 deposition observed on TKO cells in normal serum is more appropriately interpreted as a consequence of decreased antibody binding and classical pathway activation following removal of major xenoantigens, rather than as evidence of enhanced Factor H recruitment.

### 3.7 Summary and levels of evidence

Sialic acid-dependent recognition is best understood not as a single, independent rejection pathway in xenotransplantation, but rather as a functional regulatory layer that can modulate innate immune-cell responses, blood-cell homeostasis, and complement–thromboinflammatory processes. Among Siglec-related pathways, the most direct xenotransplantation evidence has been obtained for porcine Siglec-1-mediated recognition and sequestration of human erythrocytes (61, 62, 64). This represents a graft-versus-host interaction in which donor macrophages recognize and remove recipient erythrocytes. In contrast, the CD24–Siglec-10 checkpoint, Siglec-9-mediated neutrophil regulation, platelet Siglec signaling, and Factor H–sialoglycan interactions provide mechanistic rationales for how the sialome may influence innate immune and vascular homeostasis, although direct evidence that these pathways determine rejection in solid-organ xenotransplantation remains limited. Importantly, although direct receptor-level recognition of CMAH-deficient gene-edited pig RBCs by human Siglec-1 has now been demonstrated (65), whether post-editing porcine sialomes drive recipient immune-cell effector responses and *in vivo* graft injury through host-versus-graft glycan-recognition pathways still requires direct functional validation.

To clarify these differences in the levels of evidence, Table 1 summarizes the effects of major glycan gene edits on xenoantigen removal and their potential post-editing glycome consequences, whereas Table 2 compares individual glycan–receptor or glycan-recognition axes according to recognition directionality, functional consequences, evidence type, and strength. In this framework, evidence strength reflects the extent to which a proposed glycan–receptor interaction has progressed from mechanistic or receptor-level observations to receptor-dependent functional outcomes in a relevant xenogeneic system. Thus, the two tables provide complementary perspectives centered on genetic editing and mechanisms and evidence, respectively.

**Table 1:** Immunological consequences of major glycan edits beyond xenoantigen removal.

Genetic modification	Major glycan target	Established benefit	Potential post-editing glycome consequence	Potential functional consequence	Evidence level/representative references
GGTA1-KO	*α*Gal	Markedly reduces anti-αGal antibody binding and hyperacute antibody–complement injury	Redistribution of glycan precursors and changes in residual terminal glycans may occur after loss of *α*Gal synthesis	Residual non-Gal antibody recognition persists; specific effects on Siglec pathways are not established	**Established** for αGal removal; glycome remodeling supported by glycomic studies [Phelps et al., 2003 (3); Burlak et al., 2013 (32)]
CMAH-KO	Neu5Gc	Reduces anti-Neu5Gc antibody binding and further decreases human IgM/IgG recognition after GGTA1-KO	Shifts sialic acid composition from Neu5Gc toward Neu5Ac, but does not directly determine α2,3/α2,6/α2,8 linkage or generate a fully human-like sialome	May alter the repertoire of ligands available to Siglecs and other sialic acid-binding proteins; increased Factor H recruitment should not be assumed	**Established** for Neu5Gc removal; **mechanistic/incompletely defined** for downstream sialome effects [Estrada et al., 2015 (4); Tector et al., 2020 (14); Schnaar et al., 2022 (13); Lee et al., 2022 (68)]
B4GALNT2-KO	B4GALNT2-dependent Sda-like glycan	Further reduces residual non-Gal human antibody binding	Loss of the terminal GalNAc-containing branch may leave or relatively expose α2,3-sialylated precursor structures	Increased recognition by Siglec-1 or other α2,3-sialoglycan-binding receptors is biologically plausible but has not been directly demonstrated	**Established** for antibody reduction; **hypothesis** for altered Siglec recognition [Estrada et al., 2015 (4); Byrne et al., 2018 (5)]
GGTA1/CMAH/B4GALNT2 TKO	αGal, Neu5Gc, and B4GALNT2-dependent Sda-like glycans	Produces a major reduction in human IgM/IgG binding and removes the three best-established carbohydrate xenoantigen classes	Residual glycans, precursor structures, and organ- or cell-specific glycome remodeling remain; protein antigens such as SLA are also unaffected	Functional compatibility may still vary among organs and recipients, and residual glycan–receptor, complement, and cellular immune interactions may persist	**Established** for reduction of major glycan antigenicity; **incompletely defined** for the residual functional glycome [Estrada et al., 2015 (4) Martens et al., 2017 (17); Wang et al., 2019 (29)]

αGal, galactose-α1,3-galactose; Neu5Gc, N-glycolylneuraminic acid; KO, knockout; TKO, triple knockout; SLA, swine leukocyte antigen.

**Table 2:** Evidence hierarchy for glycan-dependent receptor pathways relevant to xenotransplantation.

Glycan–receptor pathway	Direction/experimental context	Potential xenotransplant consequence	Evidence type	Strength	Representative references
Porcine Siglec-1–human erythrocyte sialoglycan	Donor macrophage → recipient erythrocyte (**graft-versus-host**)	Recipient erythrocyte sequestration and erythrophagocytosis	**Direct xenotransplant**	**High**	Brock et al., 2012 (61); Waldman et al., 2013 (62); Petitpas et al., 2022 (64)
Porcine ASGR1–human platelet glycan	Donor liver lectin → recipient platelet (**graft-versus-host**)	Recipient platelet sequestration and thrombocytopenia	**Direct xenotransplant**	**High**	Burlak et al., 2010 (58); Paris et al., 2012, 2015 (59, 60)
Factor H–sialoglycan interaction	Recipient human Factor H → donor porcine cell/endothelial sialoglycan	Altered alternative complement pathway regulation	**Xenogeneic cell system**	**Moderate**	Blaum et al., 2015 (66); Schmidt et al., 2018 (67); Lee et al., 2022 (68); Teoh et al., 2023 (69)
CD24–Siglec-10 checkpoint	Recipient myeloid Siglec-10 → donor sialylated CD24-containing surface	Modulation of DAMP-driven innate immune activation	**Non-xeno mechanistic**	**Moderate**	Chen et al., 2009, 2011 (43, 45); Barkal et al., 2019 (46); Magenau et al., 2024 (79)
Siglec-9–neutrophil sialoglycan interaction	Recipient neutrophil Siglec-9 → donor sialylated ligand	Modulation of neutrophil activation and NETosis	**Non-xeno mechanistic**	**Moderate**	Lizcano et al., 2017 (47); Delaveris et al., 2021 (48)
Platelet Siglec-9 cis interaction	Recipient-intrinsic platelet cis interaction; not a donor–recipient directional pathway	Modulation of platelet activation	**Non-xeno mechanistic**	**Moderate**	She et al., 2025 (57)
Platelet Siglec-7 signaling	Platelet Siglec-7 → sialylated ligand	Modulation of platelet activation and cell-death-associated responses	**Non-xeno mechanistic**	**Low**	Nguyen et al., 2014 (56)
Human Siglec-1–gene-edited pig RBC sialoglycan	Human receptor → donor pig RBC (host-versus-graft; receptor–cell binding)	Potential donor RBC capture and erythrophagocytosis	**Xenogeneic cell system**	**Moderate**	Pham et al., 2025 (65)
CMAH/B4GALNT2 editing–dependent alteration of Siglec ligand availability	Gene-edited donor cell → recipient glycan receptor	Altered glycan-receptor recognition after xenoantigen deletion	**Inferred**	**Low**	Burlak et al., 2013 (32); Morticelli et al., 2023 (31); Büll et al., 2021 (42)

ASGR1, asialoglycoprotein receptor 1; DAMP, damage-associated molecular pattern; NETosis, neutrophil extracellular trap formation; GPIbα, glycoprotein Ib alpha. Evidence strength was operationally graded as follows: High, direct xenogeneic evidence linking a defined glycan–receptor interaction to a functional outcome with receptor/ligand perturbation or equivalent causal support; Moderate, direct receptor–cell evidence in a xenogeneic system or mechanistic functional evidence without receptor-dependent whole-organ/*in vivo* xenotransplantation validation; Low, biologically plausible or inferred evidence lacking direct receptor-dependent functional validation in a relevant xenogeneic system.

## 4 Strategies to overcome sialome and innate immune–coagulation incompatibilities in xenotransplantation

Residual barriers to xenotransplantation arise from complex interactions among antibody–complement activation, interspecies coagulation incompatibility, innate immune-cell responses, platelet consumption, and glycan–receptor incompatibility. Therefore, rather than targeting a single gene or pharmacological pathway, an integrated approach combining genetic engineering of donor pigs, recipient-side complement and coagulation control, precision glycoengineering, and *ex vivo* organ conditioning is required.

### 4.1 Optimization of multigene engineering in donor animals

The most established donor-side strategy currently combines the removal of major glycan xenoantigens with the introduction of human protective genes. For example, kidneys from donor pigs carrying 3KO together with CD46, CD55, THBD, PROCR, CD47, TNFAIP3, and HMOX1 provided life-sustaining function for up to 758 days in cynomolgus monkeys (6). This finding illustrates the importance of simultaneously optimizing xenoantigen removal and downstream effector control.

To mitigate coagulation incompatibility, human THBD, PROCR, TFPI, and CD39 have been incorporated into donor pigs, with hTHBD in particular potentially contributing to prolonged graft survival by improving activation of human protein C (70, 71). Unlike human vWF, whose A1 domain normally requires shear-dependent conformational activation for efficient engagement of platelet GPIb*α*, porcine vWF can bind human/primate GPIb*α* spontaneously under low- or no-shear conditions. This non-physiological interaction is influenced by species-specific properties of the vWF A1 domain, including differential O-linked glycosylation, and promotes platelet adhesion and activation (72, 73). Humanization of the porcine GPIb-binding region reduces this spontaneous interaction and platelet sequestration and may therefore be particularly useful in organs in which platelet consumption is prominent (73).

Reinforcement of innate immune checkpoints represents another important donor-engineering strategy. Human CD47 can reduce macrophage-mediated phagocytosis through recipient SIRP*α* signaling (74, 75). Similarly, overexpression of hCD200 in porcine endothelial cells enhances CD200R-mediated inhibitory signaling and suppresses xenogeneic phagocytosis and inflammatory responses. In a human whole-blood-perfused vascular platform and a humanized-mouse vascular xenograft model, hCD200 reduced macrophage and T-cell infiltration, IgG/C4d deposition, complement activation, platelet adhesion, and coagulation activation (76). Although hCD200 showed stronger protective effects than hCD47 for several vascular endpoints, whether these findings can be reproduced in whole-organ xenotransplantation requires further validation. Thus, the major human transgenes used in conjunction with TKO provide complementary protection at different levels of complement regulation, coagulation control, innate immune checkpoints, and cytoprotection. Their principal functions and limitations from the perspective of glycan–immune compatibility are summarized in Table 3.

**Table 3:** Donor genetic modifications that complement glycan xenoantigen removal in xenotransplantation.

Donor modification	Primary pathway/function	Expected or established benefit	Major limitation in the context of glycan–immune compatibility	Representative references
Human CD46, CD55	Complement regulation	Inhibit complement activation on the donor vascular surface and reduce complement-mediated endothelial injury	Do not remove residual xenoantigens or correct the donor sialome; antibody recognition and glycan-dependent innate immune interactions may persist	Iwase et al., 2019 (26); Anand et al., 2023 (6)
Human THBD, PROCR	Protein C anticoagulant pathway	Improve regulation of thrombin generation and reduce species-related coagulation incompatibility	Correct downstream coagulation dysregulation but do not directly prevent antibody or glycan-receptor recognition; individual porcine components may retain partial functional compatibility	Petersen et al., 2009 (70); Singh et al., 2019 (71); Salvaris et al., 2020 (77)
Human TFPI, CD39	Coagulation and platelet regulation	Limit tissue factor-dependent coagulation and extracellular nucleotide-driven platelet/endothelial activation	Provide downstream antithrombotic protection without correcting the underlying glycan or sialome mismatch	Iwase et al., 2019 (26)
Human CD47	Macrophage inhibitory signaling through SIRPα	Reduces macrophage-mediated phagocytosis of porcine cells and may improve graft protection	Restores one inhibitory checkpoint only; other macrophage recognition pathways, including antibody-, complement-, and glycan-receptor-dependent mechanisms, remain	Nomura et al., 2020 (74); Takeuchi et al., 2021 (75)
Human CD200	Inhibitory myeloid-cell signaling	May reduce macrophage activation, cytotoxicity, and inflammatory cytokine production	Additional benefit over other inhibitory transgenes is context dependent; does not directly alter donor glycan antigenicity	Sakai et al., 2018; Yan et al., 2018
Human HMOX1, TNFAIP3	Cytoprotective and anti-inflammatory regulation	Increase resistance to inflammatory and endothelial injury after immune activation	Primarily suppress downstream cellular injury and do not correct residual xenoantigens or sialome incompatibility	Iwase et al., 2019 (26); Anand et al., 2023 (6)
Humanized porcine vWF	Platelet–vWF compatibility	Reduces abnormal interaction between porcine vWF and primate platelet GPIb and may decrease platelet activation and sequestration	Addresses a specific platelet incompatibility rather than global glycan–immune mismatch; relative importance is organ dependent	Connolly et al., 2021 (73)

THBD, thrombomodulin; PROCR, endothelial protein C receptor; TFPI, tissue factor pathway inhibitor; CD39, ectonucleoside triphosphate diphosphohydrolase-1; SIRPα, signal regulatory protein α; HMOX1, heme oxygenase 1; TNFAIP3, tumor necrosis factor α-induced protein 3; vWF, von Willebrand factor; GPIb, glycoprotein Ib.

However, a larger number of human transgenes does not necessarily result in a superior donor. Some porcine molecules may retain sufficient functional compatibility with human regulatory systems (77), and the requirement for individual transgenes may differ according to organ-specific pathophysiology. Future donor design should therefore move away from simply maximizing the number of genetic modifications and instead identify the incompatibilities that are functionally relevant to each target organ and select the minimal but optimal combination of modifications required to address them.

### 4.2 Recipient-side modulation of complement, coagulation, and innate immunity

Because genetic engineering of donor pigs alone is unlikely to eliminate all interspecies incompatibilities, recipient-side control of complement and coagulation is particularly important during the early reperfusion period. C5 blockade has relatively direct evidence from pig-to-human xenotransplantation. In a brain-dead human recipient model using a 10-gene-edited pig kidney, complement control including eculizumab was associated with suppression of thrombotic microangiopathy (78). C3 inhibition has the potential advantage of blocking complement amplification at an upstream point where multiple complement pathways converge; however, direct clinical evidence for C3 inhibition in solid-organ pig-to-human xenotransplantation remains more limited than that for C5 inhibition.

Because shear-independent porcine vWF A1–human GPIb*α* engagement, tissue factor activation, and protein C pathway incompatibility may occur simultaneously, conventional anticoagulation alone is unlikely to correct all of these abnormalities. Accordingly, organ-specific combinations of donor-side coagulation engineering with recipient-side anticoagulant or antiplatelet therapy may be required.

Pharmacological modulation of Siglec pathways represents an exploratory approach that could be added to these established strategies. Siglec-9 agonism may suppress neutrophil activation and NETosis (48), while enhancement of the CD24-associated DAMP checkpoint may offer another means of controlling early sterile inflammation. Notably, the evaluation of CD24Fc in a phase 2a clinical trial in allogeneic hematopoietic stem-cell transplantation demonstrates that DAMP-directed checkpoint modulation is itself a clinically implementable strategy (79). However, this clinical precedent should not be extrapolated as evidence for the efficacy of the porcine CD24–human Siglec-10 axis. At present, such approaches are more appropriately regarded as adjunctive strategies for limiting early innate immune overactivation rather than as replacements for conventional immunosuppression or complement blockade.

### 4.3 Precision glycoengineering targeting the sialome

CMAH knockout reduces the anti-Neu5Gc antibody barrier, but the functional interactions of the post-editing Neu5Ac-dominant sialome with Siglecs and complement regulators depend on linkage, glycan carrier, density, and surface presentation. *α*2,3- and *α*2,6-linked sialylation illustrate the bidirectional trade-offs inherent in this approach. Neu5Ac*α*2,3Gal-containing structures can serve as preferred ligands for Siglec-1 while also contributing to Factor H-dependent recognition of self-associated surfaces. Conversely, increasing *α*2,6-linked sialylation may provide an immunological benefit: overexpression of *α*2,6-sialyltransferase in swine endothelial cells significantly reduced human macrophage-mediated xenocytotoxicity *in vitro*, consistent with enhanced inhibitory signaling through CD33-related Siglecs, although the responsible receptor(s) were not directly identified (80). Thus, shifting terminal sialylation toward *α*2,6 may simultaneously reduce certain Siglec-1-mediated interactions and strengthen inhibitory macrophage regulation, but could also reduce the availability of *α*2,3-linked sialoglycans that support Factor H-dependent self-surface recognition. Accordingly, global ST6GAL1-based engineering should not automatically be assumed to be uniformly protective; the goal should instead be receptor-, carrier-, cell-, and organ-specific optimization of the functional sialome.

Sialic acid modifications such as O-acetylation represent another important determinant of the functional sialome. Visser et al. reviewed evidence that O-acetylation can alter recognition by sialic acid-binding proteins and sialidases (35), whereas Vos et al. demonstrated that ion mobility–mass spectrometry can distinguish both the positions of sialic acid O-acetylation and *α*2,3-, *α*2,6-, and *α*2,8-linkages in complex biological samples (81). Such analytical approaches may be useful for characterizing genetically engineered pig organs beyond measurements of total sialic acid abundance by simultaneously defining linkage and chemical modification. Any attempt to engineer O-acetylation should therefore first establish the actual modification pattern in the target organ and subsequently determine its relationship to human Siglec and Factor H binding and downstream functional immune responses. Thus, the ultimate objective should be functional compatibility that minimizes detrimental recognition while preserving protective glycan-dependent regulation, rather than structural resemblance to the human glycome *per se*.

Genotype-level confirmation of glycosyltransferase editing should also be complemented by direct molecular verification of the resulting cell-surface glycome. Lectin staining and cellular crossmatching remain valuable screening tools but may not adequately resolve low-abundance residual epitopes, site-specific glycoforms, or post-editing microheterogeneity. Quantitative LC–MS/MS-based glycoproteomics, particularly intact glycopeptide (IGP) profiling, can simultaneously define the glycoprotein carrier, glycosylation site, and attached glycan composition. A recent antibody-enrichment/LC–MS/MS strategy identified 122 human preformed-antibody-reactive intact glycopeptides from pig kidney, including Neu5Gc-containing species, illustrating the potential of IGP profiling to connect molecular xenoantigen identity with human antibody recognition (82). For genetically engineered donor organs, quantitative comparison of WT and edited tissues using IGP profiling could therefore complement lectin and crossmatch assays to verify target-antigen depletion and detect post-editing glycoform redistribution. Because IGP analysis primarily interrogates glycoproteins, complementary glycolipid-focused glycomics may still be required for comprehensive cell-surface validation.

One potential approach to implementing such precision glycoengineering is to restrict glycosyltransferase expression to selected cell types rather than using systemic overexpression. For example, ICAM2 or Tie2/TEK regulatory elements have been used to preferentially direct transgene expression to the vascular endothelium (83–85). This raises the possibility of selectively expressing a particular sialyltransferase in donor endothelial cells to preferentially modify the glycocalyx that is directly exposed to recipient blood.

Precision sialome engineering may also include strategies to prevent the loss of pre-existing sialylation during transplantation rather than solely generating new sialic acid structures or altering glycosidic linkages. Ischemia–reperfusion injury during organ procurement, preservation, and reperfusion can damage the endothelial glycocalyx and may increase sialidase activity. In particular, NEU1 can remove terminal sialic acids from cell-surface glycoconjugates and thereby potentially alter inflammatory and vascular responses. In a myocardial ischemia–reperfusion model, NEU1 expression and neuraminidase activity were increased, whereas animals with reduced NEU1 activity showed decreased accumulation of pro-inflammatory macrophages and less subsequent cardiac dysfunction (86). In platelets, cell-surface translocation of endogenous NEU1 and desialylation of GPIb*α* can expose underlying galactose-containing termini and promote platelet-clearance signaling. These desialylated glycan termini are recognized predominantly by the hepatic Ashwell–Morell receptor (AMR; ASGR1/ASGR2), thereby facilitating platelet uptake and clearance (53, 54, 87). This NEU1–desialylation–AMR axis provides a mechanistic link by which peri-transplant loss of platelet sialylation could exacerbate thrombocytopenia, although its quantitative contribution to solid-organ xenotransplantation remains to be directly established. Accordingly, transient inhibition of NEU1 or other relevant sialidases during organ preservation or early reperfusion may represent a strategy to preserve endothelial and blood-cell sialylation.

### 4.4 Functional assessment and graft conditioning using *ex vivo* perfusion

Normothermic or subnormothermic *ex vivo* perfusion can serve not merely as a means of “adapting” donor organs to human blood, but as a platform for assessing actual hemocompatibility and organ function before transplantation and for selectively delivering therapeutic agents or gene therapy to the graft (88, 89). In particular, perfusion of pig organs with human whole blood or blood components enables functional assessment of multiple xenogeneic responses, including complement activation, platelet consumption, erythrocyte sequestration, leukocyte activation, and endothelial injury. Such models may reveal receptor-, cellular-, coagulation-, and endothelial-level incompatibilities that cannot be adequately predicted by conventional antibody-based crossmatching alone (73, 76, 90).

At the same time, *ex vivo* perfusion can provide a means for local delivery of complement inhibitors, anticoagulants, anti-inflammatory agents, and glycocalyx-protective compounds. This approach may be particularly valuable for organs such as the liver and lung, in which resident macrophage-mediated sequestration of recipient blood cells or early platelet consumption can be prominent. Future strategies could also explore selective delivery of Siglec-targeted agents or glycocalyx-modifying molecules to the graft surface, although such approaches should currently be regarded as experimental.

Accordingly, a major role of *ex vivo* perfusion can be defined as the integration of organ-specific functional crossmatching, local therapeutic intervention, and endothelial/glycocalyx conditioning within a single platform.

### 4.5 Adaptive immune regulation and immune tolerance

Even if innate immune responses and hemocompatibility are improved, regulation of adaptive immunity remains essential for long-term graft survival. In current non-human primate xenotransplantation models, immunosuppressive regimens centered on CD40–CD154 costimulation blockade have been among the key factors enabling prolonged graft survival. Accordingly, interventions targeting the sialome or Siglec pathways should be viewed as complementary to, rather than replacements for, these established immunosuppressive strategies.

In the longer term, induction of donor-specific tolerance through approaches such as thymic transplantation, mixed hematopoietic chimerism, and regulatory-cell-based therapies may provide a means to reduce the need for chronic systemic immunosuppression.

### 4.6 Organ- and recipient-specific functional monitoring

In clinical xenotransplantation, it is unlikely that pigs with the same genotype will exhibit identical immunological compatibility across all organs and all recipients. Before transplantation, recipient anti-pig IgM/IgG levels and major anti-glycan antibody profiles should be assessed, and, where feasible, crossmatching and complement-dependent cytotoxicity assays using actual donor-derived endothelial cells should be performed to define donor–recipient-specific risk.

Immediately after transplantation, serial monitoring of platelet counts, hemolysis, and markers of complement and coagulation activation may enable rapid assessment of early graft injury. In the future, these conventional parameters could be complemented by donor organ-specific sialome profiling, human Siglec and Factor H binding assays, and functional assays such as macrophage phagocytosis or neutrophil activation to provide a more precise assessment of glycan-dependent incompatibility.

Ultimately, donor selection and therapeutic intervention in xenotransplantation should evolve beyond simply asking “which genes have been edited?” toward determining how functionally compatible a given donor organ is with the blood and immune system of a specific recipient.

## 5 Discussion and future perspectives

### 5.1 Hierarchy of evidence: from direct evidence to functional causality

Current evidence indicates that glycan-dependent recognition can produce functional consequences in xenotransplantation, although the strength of evidence differs considerably among individual pathways. The strongest evidence comes from xenogeneic systems in which glycan–receptor interactions are directly linked to the capture and consumption of recipient erythrocytes or platelets. Representative examples include recipient human erythrocyte sequestration mediated by donor porcine Siglec-1 and human platelet uptake mediated by donor hepatic ASGR1 (60–62, 64). These findings demonstrate that interspecies glycan–lectin mismatches can extend beyond biochemical binding and lead to actual functional consequences in xenogeneic systems.

Evidence has also begun to accumulate for recognition in the reciprocal direction. As discussed above, direct receptor-level interactions between human Siglec-1 and CMAH-deficient gene-edited pig RBCs have been demonstrated (65), establishing Siglec-1 recognition in the host-versus-graft direction. However, the current evidence remains at the receptor–cell binding level, and it has not yet been shown whether this interaction leads to recipient macrophage phagocytosis, circulating-cell clearance, or *in vivo* graft injury.

Other pathways, including the CD24–Siglec-10 checkpoint, Siglec-9-dependent neutrophil regulation, platelet Siglec signaling, and Factor H–sialoglycan interactions, provide strong mechanistic rationales but have not yet been established as direct causal pathways in solid-organ xenotransplantation. Future studies should therefore move beyond measurements of ligand abundance or receptor binding and establish a sequential evidence chain linking (1) glycan alteration in the actual target organ, (2) specific interaction with a recipient receptor, (3) receptor-dependent immune-cell function, and (4) *in vivo* xenogeneic outcomes. Only when such an evidence chain is established can a specific post-editing sialome feature be prioritized as a direct target for donor engineering or therapeutic intervention.

### 5.2 Defining a functionally compatible sialome

An important implication of the available evidence is that a “human-compatible sialome” should not be defined simply by the degree to which the glycan composition of donor cells resembles that of human cell surfaces. Rather, functional compatibility is more appropriately defined by whether the donor glycome minimizes detrimental immune recognition while preserving protective glycan-dependent interactions.

This distinction is important because the same glycan feature can produce opposing functional consequences through different receptor systems. Future studies should therefore progress beyond descriptive glycomics toward structure-to-function analysis. Ideally, three levels of evidence should be established for each candidate glycan alteration. First, the proposed glycan structural change should be directly demonstrated in the actual target organ and relevant cell type, ideally using quantitative site- and carrier-resolved glycomic or glycoproteomic approaches rather than genotype or lectin staining alone. Second, the alteration should be shown to modify binding of the proposed human receptor. Third, a receptor-dependent functional xenogeneic phenotype should be demonstrated and shown to be reversible by receptor blockade, ligand modification, or genetic rescue.

Such an approach would allow candidate modifications to be prioritized according to their actual functional effects rather than simply by the number of genes edited. The optimal glycoengineering strategy is also likely to differ among organs. Liver sinusoidal cells, renal microvascular endothelium, cardiac endothelium, resident macrophages, and circulating blood cells possess distinct glycoconjugate landscapes and are exposed to different dominant effector mechanisms.

### 5.3 Validation strategies to bridge the translational gap

An important translational limitation is that existing non-human primate models do not fully reproduce the human glyco-immune interface. A central biological reason is the species difference in CMAH: Old World monkeys retain functional CMAH and express Neu5Gc as a self glycan, whereas humans lack functional CMAH and are Neu5Ac-dominant. Consequently, Old World monkeys are tolerant to Neu5Gc and do not reproduce the human anti-Neu5Gc antibody barrier. Conversely, CMAH deletion in donor pigs can expose or relatively increase other carbohydrate determinants against which Old World monkeys, but generally not humans, possess naturally occurring antibodies. These determinants have been operationally referred to as a “fourth xenoantigen,” although their precise molecular identity remains unresolved (25). Thus, the same CMAH deletion that improves compatibility with human serum may paradoxically worsen compatibility in Old World monkeys. Consistent with this model, Neu5Gc-negative DKO/TKO pig kidneys showed shorter survival and more frequent antibody-mediated rejection than Neu5Gc-positive GTKO kidneys in baboons (26). Therefore, post-editing glyco-immune compatibility in humans should not be inferred solely from graft survival in NHPs; human serum-, blood-cell-, and receptor-based functional assays should be incorporated to distinguish species-specific effects.

Future validation should place greater emphasis on human-based functional systems. Donor organ-specific endothelial and parenchymal cells should be evaluated in combination with human serum, whole blood, macrophages, neutrophils, platelets, and purified glycan-recognition receptors. Whenever possible, receptor blockade or genetic perturbation should be incorporated to distinguish causal mechanisms from simple associations. Whole-blood or *ex vivo* organ perfusion can then bridge reductionist assays and actual transplantation by enabling simultaneous assessment of antibody binding, complement activation, blood-cell sequestration, NETosis, platelet consumption, coagulation activation, and endothelial injury.

Such an approach may also broaden the concept of donor–recipient matching. Conventional crossmatching primarily evaluates antibody reactivity, but if specific glycan–receptor interactions are shown to predict clinically meaningful injury, a functional crossmatch incorporating selected glycan–receptor assays may become feasible.

### 5.4 Priorities for future donor design and clinical application

An immediate priority should not be the indiscriminate addition of further glycosyltransferase gene edits. New modifications should be prioritized only when a defined residual incompatibility can be linked to a specific glycan structure and, in turn, to a receptor-dependent functional phenotype. This principle favors minimal, organ-specific, and mechanistically justified donor engineering rather than increasingly complex genotypes as an end in themselves.

Sialome-directed interventions should likewise be integrated as complementary rather than replacement strategies. They should be considered in combination with removal of major xenoantigens, introduction of human complement- and coagulation-regulatory genes, reinforcement of innate immune checkpoints, and immunosuppression targeting the CD40–CD154 pathway. *Ex vivo* perfusion may be particularly useful for comparing combinations of donor genotypes and local interventions before transplantation and for identifying organ-specific vulnerabilities that cannot be predicted from genotype alone.

Ultimately, the clinical relevance of the sialic acid–Siglec axis will depend on demonstrating that modulation of a defined glycan–receptor interaction improves meaningful xenotransplantation endpoints. Until such evidence is established, this axis is best regarded not as an alternative explanation for established rejection mechanisms, but as a candidate regulatory layer that may account for part of the residual innate immune and thromboinflammatory incompatibility that persists after removal of major xenoantigens.

### 5.5 Conclusion

Removal of major carbohydrate xenoantigens has dramatically advanced the feasibility of pig-to-human transplantation, but antigen deletion alone does not ensure complete glyco-immune compatibility. Although several direct glycan-dependent xenogeneic interactions have now been demonstrated, many mechanisms involving the sialic acid–Siglec axis remain supported primarily by mechanistic or inferential evidence. The next step is therefore to directly connect organ-specific glycan structures with defined human receptors and receptor-dependent functional outcomes. Such evidence-based functional glycoengineering may provide a means to further refine the compatibility of genetically engineered xenografts without overstating the current role of the sialic acid–Siglec axis.
